# Understanding the rules of the road: proteomic approaches to interrogate the blood brain barrier

**DOI:** 10.3389/fnins.2015.00070

**Published:** 2015-03-04

**Authors:** Bruce E. Torbett, Andrew Baird, Brian P. Eliceiri

**Affiliations:** ^1^Molecular and Experimental Medicine, The Scripps Research InstituteLa Jolla, CA, USA; ^2^Department of Surgery, University of California, San DiegoSan Diego, CA, USA

**Keywords:** blood brain barrier, proteomics, mass spectrometry, vascular diseases

## Abstract

The blood brain barrier (BBB) is often regarded as a passive barrier that protects brain parenchyma from toxic substances, circulating leukocytes, while allowing the passage of selected molecules. Recently, a combination of molecular profiling techniques have characterized the constituents of the BBB based on *in vitro* models using isolated endothelial cells and *ex vivo* models analyzing isolated blood vessels. Characterization of gene expression profiles that are specific to the endothelium of brain blood vessels, and the identification of proteins, cells and multi-cellular structure that comprise the BBB have led to a emerging consensus that the BBB is not, in and of itself, a simple barrier of specialized endothelial cells. Instead, regulation of transcytosis, permeability, and drug translocation into the central nervous system is now viewed as a collection of neurovascular units (NVUs) that, together, give the BBB its unique biological properties. We will review recent technology advancing the understanding of the molecular basis of the BBB with a focus on proteomic approaches.

The focus of this review is on techniques that can be applied to the analysis of *in vivo* models with particular attention to the complementary value of transcriptomics (Enerson and Drewes, 2006; Daneman et al., 2010) where sensitivity and standardized analytical techniques in RNA expression are powerful tools that can provide optimal coverage of gene expression in select tissues and cell types. Furthermore, we address the challenges of analyzing the protein composition of the BBB from the perspective of not only the endothelial cells, a compartment that has been the focus of *in vitro* models, but also the importance of considering the BBB as a multicellular structure with extracellular matrix (ECM) and other cell types relevant in BBB formation (Chun et al., 2011; Hoshi et al., 2013; Badhwar et al., 2014).

## Proteomics for blood brain barrier interrogation

Proteomics, the global interrogation of the protein population expressed by a genome, cells, or tissue types, makes use of biochemical and physical methods to determine the identity of proteins present (Yates, 1998, 2000; Banks et al., 2000; McDonald and Yates, 2000; Pandey and Mann, 2000; Yates, 2000; Mann and Pandey, 2001; Mann et al., 2001). Proteomics is complementary to studies of gene expression. Determination of the changes in cellular mRNA abundance provides information on gene activity and the cellular state (Enerson and Drewes, 2006; Daneman et al., 2010). For many genes, changes in mRNA abundance correspond to changes in protein abundance (Yates, 1998; Banks et al., 2000; McDonald and Yates, 2000; Pandey and Mann, 2000; Yates, 2000; Mann and Pandey, 2001; Mann et al., 2001). However, protein-based cellular analysis is essential to establish the translation of mRNAs to proteins, since mRNAs have varying stability, translational efficiency and do not address post-translational modifications. The diverse chemical properties of proteins make them difficult to separate and identify. Two-dimensional gel electrophoresis has been the standard for separation, isolation, and sequencing of individual proteins from sera, cells, and tissues for more than 25 years. However, this methodology does not provide high throughput protein identification and quantification required for the study of the cellular proteome. What revolutionized the proteomic field was the development of mass spectrometry instrumentation capable of rapidly and reproducibly fragmenting peptides and bioinformatic software to match the observed peptide fragment masses to a database of predicted masses for one of many given peptide sequences (Washburn et al., 2001; Wu et al., 2003).

Shotgun proteomics or Multi-Dimensional Protein Identification Technology (MudPIT) is the contemporary approach for identifying proteins in complex cellular samples (Washburn et al., 2001; Wu and Maccoss, 2002; Wu et al., 2003; Kislinger et al., 2005; Yates et al., 2009). The methodology relies on protease digestion of the cellular protein sample to peptides, followed by peptide separation on inline high performance chromatography, and identification of the peptides by tandem mass spectrometry (Chun et al., 2011; Hoshi et al., 2013; Badhwar et al., 2014). The uniqueness of the mass fragmentation of each peptide is used to identify the protein from which it was derived (Chun et al., 2011; Hoshi et al., 2013; Badhwar et al., 2014). The advantage of MudPIT, over classical two dimensional gel analyses, is the better resolving power, enabling a more precise protein quantification and identification of a larger number of proteins from the complex proteome of isolated tissues or cells (Washburn et al., 2001; Aebersold and Mann, 2003; Wu et al., 2003; Pottiez et al., 2009). In addition to being useful for tissues, such techniques are sensitive enough for use on desired cell populations isolated by flow cytometry or magnetic beads. Importantly, the increasing resolving power of the mass spectrometers, highly curated and annotated databases for protein identification, and bioinformatic to identify proteins from databases, provides the researcher with powerful methods to analyze complex cellular protein mixtures (Chen et al., 2006; Ruse et al., 2008; Gonzalez-Begne et al., 2009).

In contrast to transcriptomics methodology, which takes advantage of chemical similarity among RNAs allowing quantification among experimental samples, quantification of proteins in complex protein samples are hindered by the diverse chemical nature of proteins and the resolving power of mass spectrometers (Coombs, 2011). One method for relative protein quantification from different experimental samples (i.e., control vs. tests) in an experimental study relies on a label-free approach of determining peptide abundance through spectral counting in each sample, thereby allowing comparison among the experimental samples (Florens et al., 2006; Mueller et al., 2008; Neilson et al., 2011; Nahnsen et al., 2013). A more precise method for determining the relative abundance of proteins from experimental samples is to label proteins in each experimental sample with a unique stable isotope, thereby allowing the mass spectrometer to identify identical proteins with different masses (McDonald and Yates, 2000; Wu et al., 2004; Gouw et al., 2008; Liao et al., 2008; Uchida et al., 2013). The differentially isotope labeled samples are combined and analyzed together and the relative abundance of each protein in the mixture can be determined. Examples of these methods are Stable Isotope Labeling with Amino acids in cell Culture (SILAC), which relies on labeling of proteins in cellular samples by metabolic labeling with ^15^N where the differential in ^15^N/^14^N labeling can be used to quantify the relative abundance of identical proteins grown under different conditions (Wu et al., 2004; Lu et al., 2007; Gouw et al., 2008; Haqqani et al., 2008; Kamiie et al., 2008; Liao et al., 2008; Evans et al., 2012). This approach and other related metabolic cell labeling technologies are powerful tools where the model system, either cell culture or an animal, can be metabolically labeled to enable absolute quantification based on the relative uptake of labeled isotopes. The application of these approaches for the annotation of protein and nucleotide databases underscores the importance of multidisciplinary technologies to better understand correlations between gene expression and protein translation (Mann and Pandey, 2001; Mann et al., 2001; Ohtsuki et al., 2011; Uchida et al., 2013). Additional quantitative techniques that have been previously reviewed include labeling of a peptide mixture with N-terminal or reactive amines using defined molecular tags, termed Isobaric Tags for Relative and Absolute Quantitation (iTRAQ) and Tandem Mass Tag (TMT) (Wu et al., 2004; Gouw et al., 2008; Christoforou and Lilley, 2012; Evans et al., 2012; Geillinger et al., 2012; Ivancic et al., 2013).

## Protein prioritization based on functional interactions between capillaries, glia, neurons, and the basal lamina

**Figure 1:**
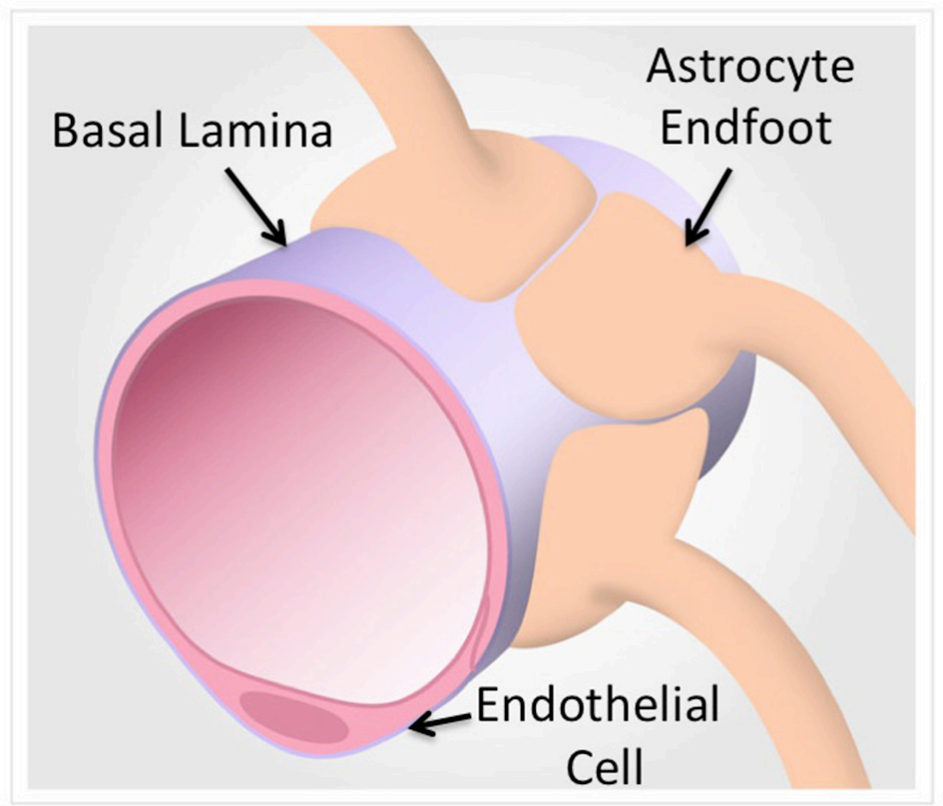
**A model of the neurovascular unit in capillaries**. Proteomic technologies that focus on the protein components of brain endothelial cells and associated astrocytes from intact capillaries identify basal lamina components that are under-represented in purified cells (Chun et al., 2011). In contrast, Proteomic analyses of purified cells yields quantitative and definitive cell type specific protein expression (Reviewed in Table 1).

The BBB is generally regarded as a passive barrier that protects brain parenchyma from toxic substances. Vascular biologists studying BBB function have often ignored the neuronal components present in freshly isolated tissues and turned to endothelial cell models separated from the endogenous neuronal-astrocytic milieu. In some cases, cell models were derived from different cell types altogether to represent the BBB. However, over the course of the last several years, a general consensus has emerged that the BBB is not a simple barrier of specialized endothelial cells controlling transcytosis, permeability and drug translocation into the CNS (Neuwelt et al., 2008; Abbott, 2013) (Figure 1). Rather, the BBB is now correctly viewed as a collection of neurovascular units (NVUs) that, together, give the BBB its unique features (Abbott et al., 2006; Cecchelli et al., 2007; Hermann and Elali, 2012; Elali et al., 2014). NVUs are localized throughout the CNS. In larger microvessels (e.g., arterioles), the NVU is composed of endothelial cells, Aquaporin 4-positive (AQP4^+^) feet vs. GFAP positive (GFAP^+^) astrocytes, and neuronal projections. These interactions can regulate vasodilation in arterioles (i.e., neurovascular coupling) in contrast to capillary endothelial cells that have no direct neuronal projections (Simard et al., 2003). A more precise description of the NVU requires consideration of the direct interactions of AQP4^+^ GFAP^−^ astrocyte end-feet upon capillary endothelial cells (Chun et al., 2011). These AQP4^+^ GFAP^−^ astrocytes are an important class of astrocytes that are in contact with a basal lamina that envelopes the outer surface brain capillary (Muldoon et al., 1999; Simard et al., 2003). Furthermore, the isolation and analysis of intact microvessels from mouse brain identifies extracellular matrix (ECM) proteins expressed in the blood vessel microenvironment, many of which are known to be basal lamina proteins (Chun et al., 2011). Table 1 summarizes the approaches for isolation of blood vessels for proteomics studies where the focus is on the analysis of isolated brain blood vessels. These proteomic data sets allow for comparisons with data sets obtained from cultured brain endothelium, where the absence of ECM microenvironment and supporting cells of the NVIU limits the translation to physiological context. We propose that this model of the NVU should be front and center for prioritization of analysis of proteins identified by proteomic technologies. Specifically, physiological context can be used to categorize various proteins that may be identified as a consequence of increased/decreased BBB integrity based on the presence of various specific cell types and their response to local or distal stimuli.

**Table 1:** **List of proteomic studies using isolated brain blood vessels**.

**Species**	**Source**	**Isolation method**	**Analytical method**	**References**
Human	Microvessels	Density gradient	LC-MS/MS with *in silico* selection	Uchida et al., 2011
Mouse	Microvessels	Glass bead	LC-MS/MS MudPIT	Chun et al., 2011
Mouse	Microvessels	Glass bead	Tag labeling/LC-MS/MS	Uchida et al., 2013
Mouse	Artery	Microdissection	LC-MS/MS MudPIT	Badhwar et al., 2014
Rat	Vessels	Laser capture	ICAT with LC-MS/MS	Haqqani et al., 2005
Mouse	Vessels	Laser capture	Gel/Fourier transform MS	Murugesan et al., 2011
Marmoset	Microvessels	Density gradient	Tag labeling/LC-MS/MS	Hoshi et al., 2013
Rat	Microvessels	Density Gradient	Tag labeling/LC-MS/MS	Hoshi et al., 2013

## Proteomics approaches based on isolated blood vessels

Since the BBB is in fact a multicellular structure, with essential ECM contributions to the organization and maintenance of the BBB integrity, arguably it is the proteomic analyses of intact brain blood vessels from human, rat, and mouse where the significant growth opportunity is present for the BBB proteomics community in the coming years. As with any molecular profiling technology, attention to the technical details, and quality control of the isolation method are critical to the relevance of the data sets obtained. For example, the emergence of multiple techniques from different groups has enabled the cross-comparison between mouse capillaries and mouse arteries to assess differences in the vascular phenotypes in the brain that have distinct biological functions (Enerson and Drewes, 2006; Daneman et al., 2010; Chun et al., 2011; Ohtsuki et al., 2013; Badhwar et al., 2014). For example, the emerging importance of extracellular microvesicles in intercellular communication in BBB biology has been recently addressed with proteomics, advancing the understanding of the composition of an organelle that is under-represented in the BBB literature (Simpson et al., 2008; Haqqani et al., 2013). Furthermore, these studies have provided specific methods that cross-validate the methods used for vessel isolation and proteomic analysis.

In the study by Chun et al. (2011), the focus was on the identification of membrane proteins and ECM proteins. The characterization of membrane proteins from isolated blood vessels vs. cultured endothelial studies enables comparison of proteins between intact *in vivo* models and more established, homogenous and quantitative assays *in vitro* using cultured endothelial cells. Expression of membrane transporter proteins validates the identification of ECM proteins that comprise the basal lamina, a class of proteins that is otherwise generally under-represented in RNA studies of microvessels, likely different from the ECM proteins expressed in endothelial cells cultured on plastic. Finally, the report on cell type-specific RNA expression in endothelial cells vs. associated astrocytes (Daneman et al., 2010) enables the classification of proteins based on the relative expression of the RNA that likely predicts protein expression. So while RNA expression is an imperfect assessment of protein expression profiles, the alternate proteomic techniques are complementary. The application of MudPIT to the analysis of primary mouse brain microvessels, isolated from the mouse cortex using the glass bead technique (Hartz et al., 2006; Yousif et al., 2007), established a mouse protein expression resource that can be compared with other methods. Chun et al. (2011), based their original approach on the initial work of Enerson and Drewes (2006) in the characterization of the isolated rat microvessel transcriptome, which was used as a foundation for the cross-comparison with transcriptome of isolated endothelial cells and astrocytes, and protein expression of transporters (Chun et al., 2011).

## Vessel heterogeneity

In the recent study by Badhwar et al. (2014), mouse brain arteries were dissected from the circle of Willis (CW) structure formed at the confluence of the internal carotid arteries, a portion of the vertebrobasilar artery and branching arteries. The goal of this study was to determine the protein expression profile of a CW, a vascular structure that is critical to the perfusion of deep cortical regions of the brain, which are affected by aging and neurodenegerative disorders associated with changes in vessel wall thickness and elasticity. The microsurgical isolation of the CW arteries in the mouse provides for a highly enriched starting material for MudPIT analysis, with a comparatively homogenous vascular phenotype. In this study both gel-based and gel-free (i.e., strong cation exchange chromatography) followed by tandem mass spectrometry were performed to obtain a comprehensive resource for mouse CW arterial vessels (Badhwar et al., 2014). Proteins identified by MudPIT were validated by Western blot analysis and exhaustively compared with the mouse microvascular proteome published by Chun et al. (2011), and were annotated with common and unique proteins in each dataset listed (Badhwar et al., 2014). The PANTHER classification identified proteins as blood-brain barrier-specific cell type proteins, tight junction and adhesion proteins, membrane transporter and channel proteins, and ECM and basal lamina proteins (Mi et al., 2013). In each case identified proteins were verified for their detection in previously reported mouse and rat transcriptome studies. Protein components related to signaling pathways, vasoactivity, arterial proteins, and neuronal proteins and were also identified.

## Proteomic analyses in disease and animal models

The cross-comparison of this arterial vessel resource with microvessels provides an important database in which to analyze the effects of genetic mutants and (Agarwal et al., 2012) injury/tumor models that impact cerebrovascular biology. As more proteomic studies are completed the more comprehensive the datasets will be for the researcher. Based on the isolation of intact brain blood vessels from mice, recently characterized mouse mutant studies can be further analyzed and compared with isolation techniques such as laser capture microdissection, and analytical techniques based on gel electrophoresis, and cultured endothelial cells. Laser microdissection enables the isolation of discrete structures enriched in specific cell types that can be analyzed for gene expression with a high degree of sensitivity as well as proteomics using mass spectrometry (Haqqani et al., 2005; Murugesan et al., 2011). The power of such techniques complements the isolation of microvessels and arteries and provides opportunities to further characterize knockout mouse models, such as the P-gp/Bcrp mouse model (Agarwal et al., 2009, 2012).

Lastly, the challenges of determining differences in rodent vs. primate protein constituents of the blood brain barrier (BBB) have been recently addressed by Hoshi et al. (2013), where isolated blood vessels from rats and marmosets were subjected to MudPIT using a quantitative approach based on an *in silico* peptide selection. In this model, side-by-side comparison of rat vs. marmoset data sets with human proteins related to endothelial cell-related membrane transporters, receptors and tight junction proteins revealed several interesting insights. Significant 2 fold differences in several key transporters were observed between rat and human blood vessels (Hoshi et al., 2013). Interestingly few significant changes in transporters between human and marmoset blood vessels were observed, suggesting that marmosets may be a useful tool for studying select brain blood vessel proteins that are substantially different between rodents and humans (Hoshi et al., 2013). This study is an excellent example of the power of the quantitative approach to define more precisely the expression profile of cell membrane transporters that have relevance to drug development.

As the methodology and bioinformatics improve for both proteomics and transcriptomics it may soon be possible to correlate relative levels of gene expression with protein changes (Wang et al., 2014). The focused application of such technologies will enable a more complete understanding of the role of secreted factors (i.e., exosomes, growth factors) and how these components may condition the basal lamina and signal to associated cells in disease.

### Conflict of interest statement

The authors declare that the research was conducted in the absence of any commercial or financial relationships that could be construed as a potential conflict of interest.

## Abbreviations

BBB: Blood brain barrier
NVU: neurovascular unit
MudPIT: multi-dimensional protein identification technology
ECM: extracellular matrix
iTRAQ: Tags for Relative and Absolute Quantitation
TMT: Tandem Mass Tags
AQP4: Aquaporin 4
GFAP: glial fibrillary acidic protein
CW: circle of Willis.
